# Lipid Droplet Motility Increases Following Viral Immune Stimulation

**DOI:** 10.3390/ijms22094418

**Published:** 2021-04-23

**Authors:** Ebony A. Monson, Donna R. Whelan, Karla J. Helbig

**Affiliations:** 1 Department of Physiology, Anatomy and Microbiology, School of Life Sciences, La Trobe University, Melbourne 3086, Australia; e.monson@latrobe.edu.au; 2 Department of Pharmacy and Biomedical Sciences, La Trobe Institute for Molecular Science, La Trobe University, Melbourne 3086, Australia

**Keywords:** lipid droplet, organelle, virus, immune, motility

## Abstract

Lipid droplets (LDs) have traditionally been thought of as solely lipid storage compartments for cells; however, in the last decade, they have emerged as critical organelles in health and disease. LDs are highly dynamic within cells, and their movement is critical in organelle–organelle interactions. Their dynamics are known to change during cellular stress or nutrient deprivation; however, their movement during pathogen infections, especially at very early timepoints, is under-researched. This study aimed to track LD dynamics in vitro, in an astrocytic model of infection. Cells were either stimulated with a dsRNA viral mimic, poly I:C, or infected with the RNA virus, Zika virus. Individual LDs within infected cells were analysed to determine displacement and speed, and average LD characteristics for multiple individual cells calculated. Both LD displacement and mean speed were significantly enhanced in stimulated cells over a time course of infection with an increase seen as early as 2 h post-infection. With the emerging role for LDs during innate host responses, understanding their dynamics is critical to elucidate how these organelles influence the outcome of viral infection.

## 1. Introduction

Lipid droplets (LDs) are ubiquitous organelles that were originally thought of as only lipid storage compartments for cells; however, in the last decade they have emerged as critical organelles in health and disease. LDs consist of a neutral lipid core of triglycerides and sterol esters that are surrounded by a monolayer of phospholipids, and originate from the endoplasmic reticulum (ER), where they bud off from the ER into the cytoplasm (reviewed in [1]).

LDs are highly motile within the cytoplasm of cells [2,3] and, like other intracellular organelles, LDs can display a variety of states of motion. The two most common forms of motion reported for LDs are Brownian diffusion within a confined space (e.g., around a tether point, such as the ER), and directed motion along linear tracks (e.g., microtubules) [4]. Brownian motion is the irregular movement of particles through a liquid or gas as influenced by random collisions with other particles and the viscosity of the surrounding liquid medium [5,6]. In the living cell, any true Brownian motion of organelles is significantly restricted by further steric hindrances and strong interactions [7]; for example, LDs tethered to the ER. Directed motion of organelles can be enhanced by ATP-dependent activity, such as molecular motors acting on the cytoskeleton [8,9]. Individual LDs can also switch between these two motility states; however, the mechanism and reason for this switching is currently not known [4]. Owing to their ability to move around a cell, LDs can physically and functionally interact with many cellular organelles, including the ER, mitochondria, peroxisomes, lysosomes and other LDs (reviewed in [10]). LD–inter-organelle interactions are critical for several processes within a cell; for example, LD–mitochondria interactions as well as LD–peroxisome interactions are required for efficient catabolism of fatty acids [11,12,13] and LD–ER contacts are important for the regulation of lipid storage and growth of LDs [14,15]. LD–organelle interactions also promote the transfer and/or the activation of a wide range of proteins potentially affecting multiple signalling pathways (reviewed in [10]). 

Recent findings from several groups suggest that the dynamics of cellular organelle contacts is in part controlled by the motility of LDs, due to the redistribution of LDs between clustered and dispersed states, resulting in altered organelle contacts (reviewed in [16]). It is known that LDs change their distribution depending on the physiological state of the cell, with LDs most notably changing distributions in times of nutrient abundance or deprivation, and under cellular stress conditions (reviewed in [17]). Interestingly, several intracellular pathogens can also induce LD rearrangement in cells [18,19]. Infection with the bacterium *Chlamydia trachomatis* induces the translocation of host LDs into the vacuole [18], whereas infection with hepatitis C virus causes LDs to aggregate near the microtubule-organising centre in a dynein-dependent redistribution, with rotavirus also causing redistribution of LDs following cellular infection [19,20].

Pathogens such as bacteria, parasites and more recently viruses, are well known to induce LD accumulation upon host cell infection [21] (Reviewed in [22]). This accumulation of LDs has been shown to drive an enhanced immune response in respect to viral infection, and to alter LD–mitochondrial interactions which underpin metabolic changes in the cell [21,23,24]. Additionally, it has recently been demonstrated that the LD resident protein, viperin, interacts with STING, likely on the Golgi and the ER, as well as TRAF6 and IRAK1 in the TLR7/9 pathways [25,26]. It is possible that the LD is involved in multiple organelle interactions that may facilitate and underpin a successful antiviral response following cellular infection. To date, there have been no studies examining the motility of LDs in cells following viral infection, or, indeed, following activation of early innate immune signalling pathways. In this research we sought to address this knowledge gap by examining changes in LD motility following the viral infection of mammalian cells at early timepoints. 

## 2. Results

### 2.1. Development of an Imaging and Analysis Pipeline to Track Lipid Droplets in Live Cells

**Figure 1:**
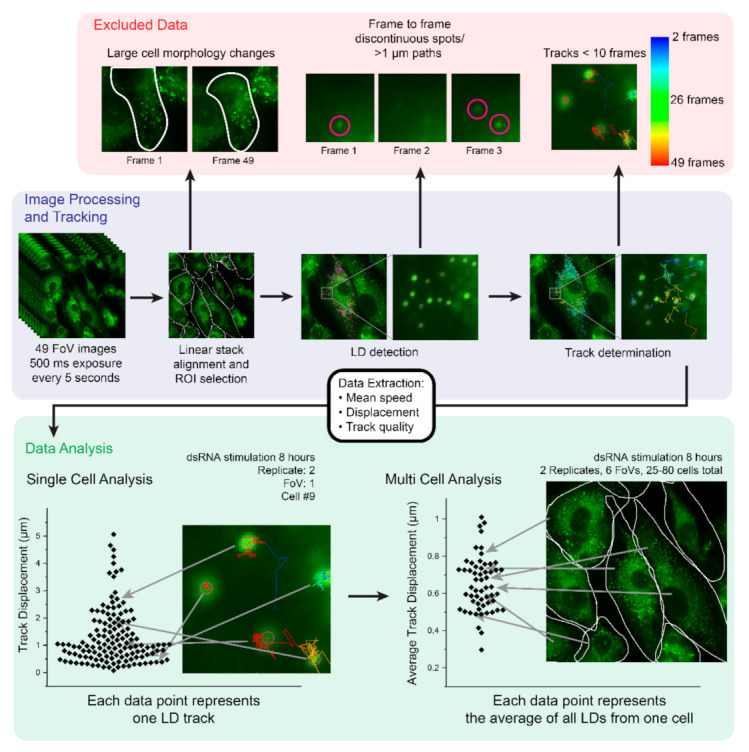
Schematic of the analysis pipeline. Primary immortalised astrocytes were stained live with Bodipy (493/503) to visualise LDs prior to imaging for LD movement in cells. A total of 49 FoV images were captured at 500 ms exposure rates every 5 s. Image stacks were aligned using linear stack alignment with SIFT plugin and ROIs were drawn around single cells in an FoV. LDs were tracked using TrackMate plugin on FIJI software where mean speed, displacement and track quality were extracted and imported into Excel. Data was cleaned up by the exclusion of any data with large cell morphology shifts, >1 µm gaps/missing frames in LD paths and tracks which were < 10 frames. Data was graphed as multi-cell analysis with each data point representing the average of all LD tracks within a single cell.

To examine LD motility dynamics in the context of the activation of early innate immune pathways, we first developed a pipeline to image and track LDs in live primary immortalised astrocyte cells. Astrocytes were chosen as a model cell type, because they are known to alter LD numbers following viral stimulation and infection, and have a high number of basal LDs [21]. Additionally, astrocytes are known to be rapid producers of an effective antiviral response [27]. Astrocytes cultured in 35 mm glass bottom dishes were labelled with Bodipy (493/503) to stain for LDs. A total of 49 time-lapse images over six fields of view (FoV) were captured using fluorescence microscopy (500 ms exposure, images captured every 5 s) (Figure 1). Images were imported into FIJI analysis software and registered using the linear stack alignment with SIFT (scale invariant feature transform) plugin [28]. Regions of interest (ROIs) were manually drawn around individual cells in each of the six FoVs before LDs within each ROI were tracked using TrackMate software on FIJI [29]. Mean speed, displacement and track quality were extracted for further processing (Figure 1). All captured data was initially screened to exclude cells that had undergone large morphology changes (i.e., dying cells), when LDs were detected but were discontinuous (i.e., >1 µm movement between frames, spots missing in sequential frames) and to exclude LD tracks that lasted less than 10 sequential frames. The exclusion of this data ensured that false positive tracks were not included in downstream data analysis (Figure 1). Individual LD tracks could be analysed or further processed to produce average displacement and speed values for each individual cell, allowing for multi-cell analysis. This was performed to generate a general picture of the LD dynamics within cells, and to remove the potentially confounding nature of extreme outliers (Figure 1).

### 2.2. Analysis of Cell-to-Cell Variability

**Figure 2:**
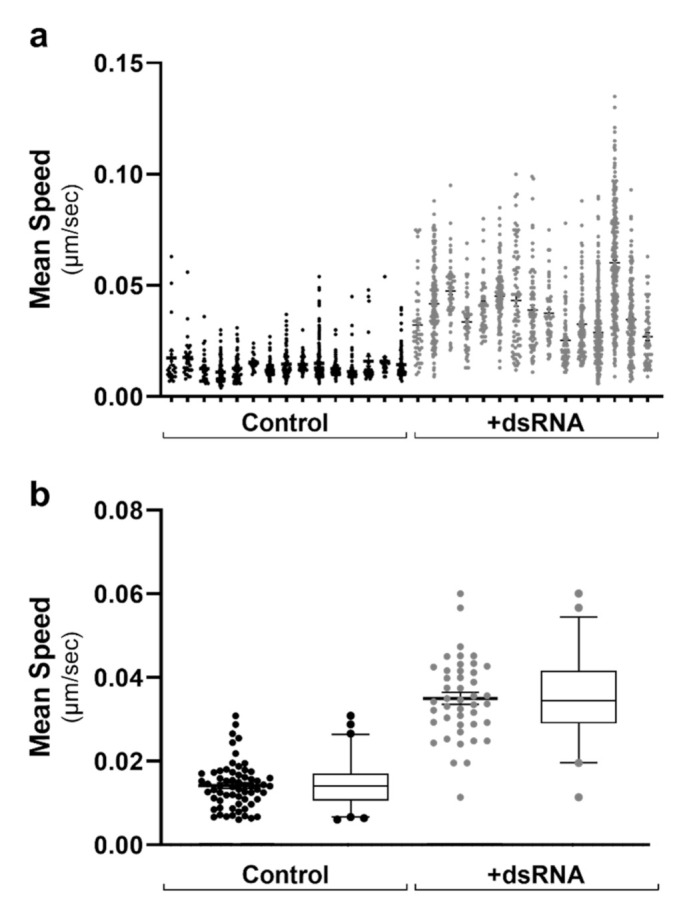
Lipid droplet tracks graphed as multi-cell analysis for downstream comparisons. Following the live image acquisition of LD motility, the mean speed of LD tracks were graphed as (**a**) single-cell analysis of individual tracks within each cell (*n* = > 20,000 tracks). Data is represented as a scatter plot with individual points. As there were *n* > 40 individual cells within each condition, data was condensed as (**b**) multi-cell analysis with all tracks within an individual cell averaged to a single data point. Data is represented as both scatter plots and box and whisker plots showing the mean, with 25–75 percentile range as the box and 5–95 percentiles as the whiskers. Control: *n* = 60 RNA: *n* = 41 cells.

**Figure 3:**
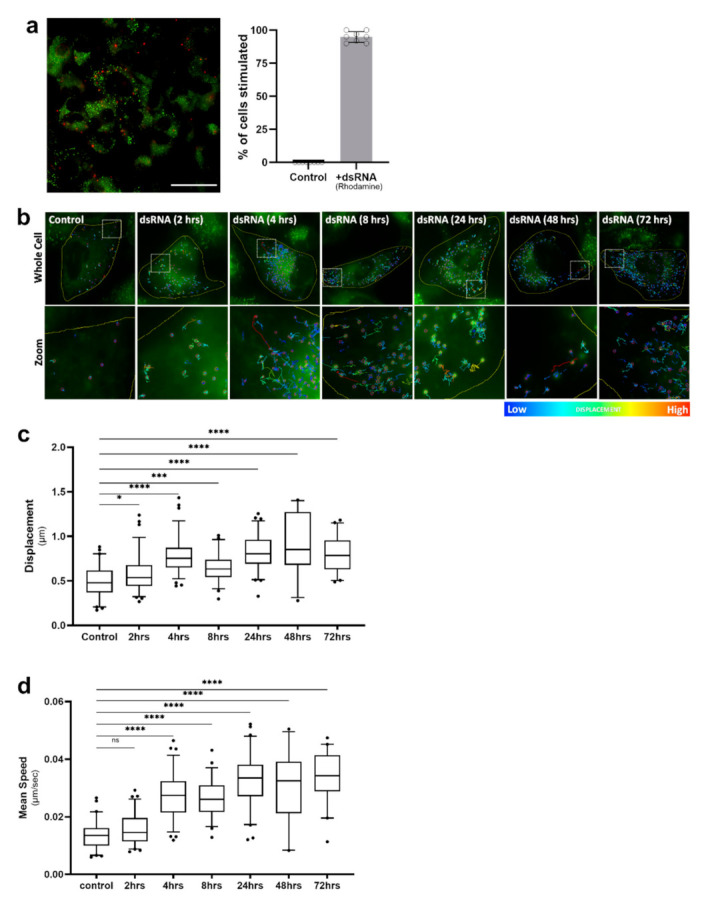
Lipid droplet displacement was increased following dsRNA viral mimic stimulation. Primary immortalised astrocyte cells were stained live with Bodipy (493/503) to visualise LDs and stimulated with fluorescent dsRNA-Rhodamine for a series of indicated timepoints prior to imaging for LD movement in cells for 240 s (49 frames). (**a**) Representative of cells that had been stimulated with the fluorescent dsRNA-Rhodamine and percentage of cells which received the dsRNA stimulant (*n* = 100 cells across 5 fields of view over 2 biological replicates), scale bar, 50 μM. (**b**) Tracks demonstrating LD displacement over a time course of dsRNA stimulation with (**c**) analysed data summary of LD displacement and (**d**) average LD speed with individual cells averaged to a single data point. Data is represented as box and whisker plots showing the mean, with 25–75 percentile range as the box and 5–95 percentiles as the whiskers. The number of cells varied for each condition due to the exclusion of some data, therefore control: *n* = 60, 2 h: *n* = 78, 4 h: *n* = 76, 8 h: *n* = 57, 24 h: *n* = 66, 48 h: *n* = 27, 72 h: *n* = 41 cells. *n* = 6 replicate movies over 2 biological replicate samples, *n* > 20,000 individual LD tracks. Data was analysed using a one-way AVOVA with Dunnett’s multiple comparisons test **** = *p* < 0.0001, *** = *p* < 0.001, * = *p* < 0.05, ns = not significant.

**Figure 4:**
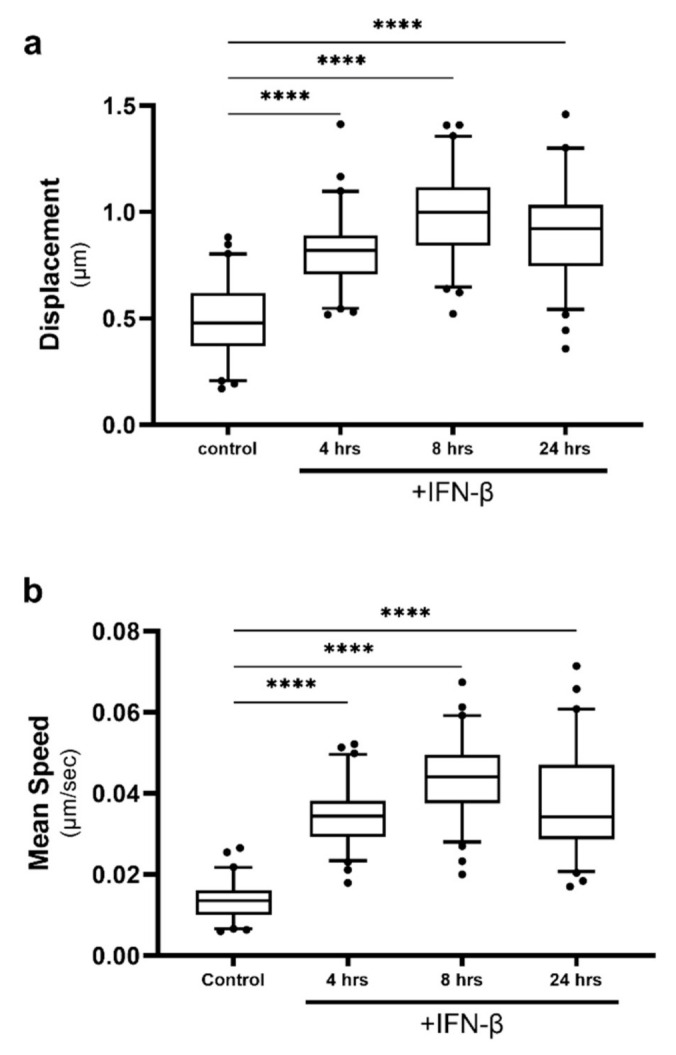
Lipid droplet speed and displacement was increased following IFN stimulation. Primary immortalised astrocyte cells were stained live with Bodipy (493/503) to visualise LDs and stimulated with IFN-β at 1000 U/mL. Cells were imaged for LD movement at 4, 8 and 24 h for 240 s (49 frames). (**a**) Average LD displacement and (**b**) average LD speed post-IFN-β stimulation was analysed (at 4, 8 and 24 h). Data is represented as box and whisker plots showing the mean, with 25–75 percentile range as the box and 5–95 percentiles as the whiskers. The number of cells varied for each condition due to the exclusion of some data, therefore control: *n* = 60, 4 h: *n* = 65, 24 h: *n* = 65, 48 h: *n* = 65. *n* = 6 replicate movies over 2 biological replicate samples, *n* > 20,000 individual LD tracks. Data was analysed using a one-way AVOVA with Dunnett’s multiple comparisons test **** = *p* < 0.0001.

**Figure 5:**
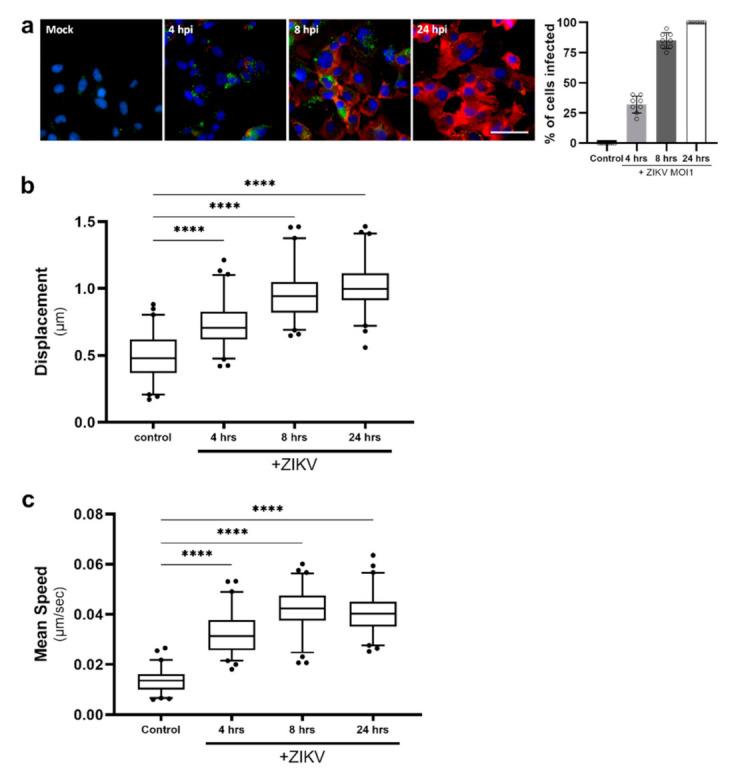
Lipid droplet speed and displacement was increased following ZIKV infection. Primary immortalised astrocyte cells were stained live with Bodipy (493/503) to visualise LDs and infected with ZIKV (MR766 strain) at an MOI1 for 4, 8 and 24 h, and were imaged for LD movement in live infected cells for 240 s (49 frames). (**a**) Following live imaging, cells were fixed and stained with Bodipy (409/505) to visualise LDs and DAPI to visualise the cell nuclei. ZIKV RNA was detected using an anti-3G1.1 and 2G4 dsRNA antibody. *n* = 100 cells across 5 fields of view over 2 biological replicates, scale bar, 50 μm. (**b**) Average LD displacement and (**c**) average LD speed post-ZIKV infection was analysed (at 4, 8 and 24 h). Data is represented as box and whisker plots showing the mean, with 25–75 percentile range as the box and 5–95 percentiles as the whiskers. The number of cells varied for each condition due to the exclusion of some data, therefore control: *n* = 60, 4 h: *n* = 64, 24 h: *n* = 68, 48 h: *n* = 60. *n* = 6 replicate movies over 2 biological replicate samples, *n* > 20,000 individual LD tracks. Data was analysed using a one-way AVOVA with Dunnett’s multiple comparisons test **** = *p* < 0.0001.

**Figure 6:**
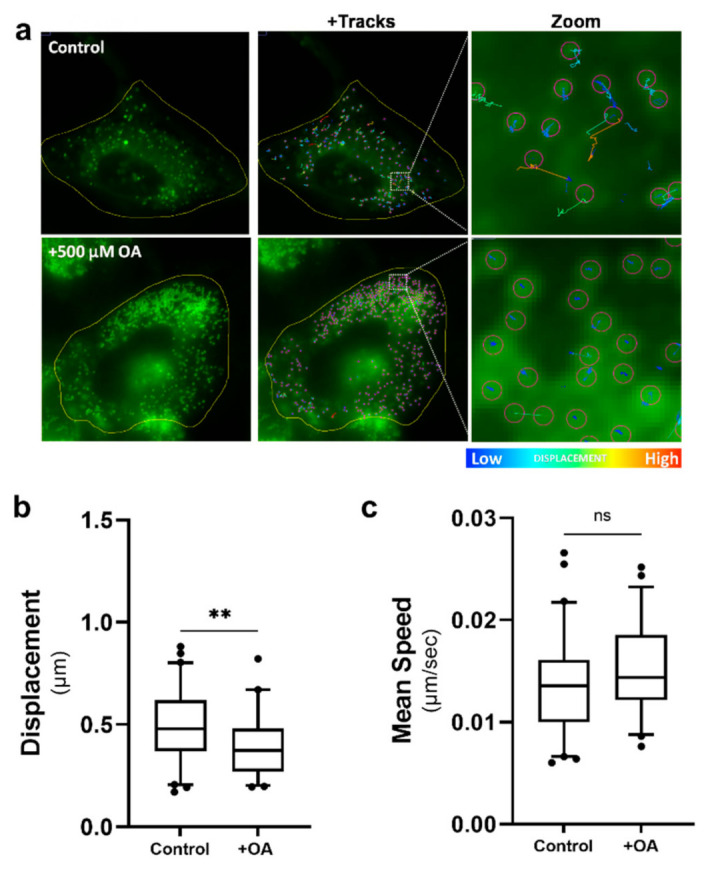
Lipid droplet speed and displacement was not controlled by increasing the number of lipid droplets. Primary immortalised astrocyte cells were treated with 500 μM oleic acid for 16 h, prior to live staining with Bodipy (493/503) to visualise LDs and imaged for LD movement for 240 s (49 frames). (**a**) Representative cells prior to and following treatment with 500 µM oleic acid (OA). Tracks demonstrating that there was not an increase in the motility of LDs following OA treatment. (**b**) Average LD displacement and (**b**) average LD speed post-OA treatment were analysed 16 h post-treatment. Data is represented as box and whisker plots showing the mean, with 25–75 percentile range as the box and 5–95 percentiles as the whiskers. The number of cells varied for each condition due to the exclusion of some data, therefore control: *n* = 60, OA: *n* = 46. *n* = 6 replicate movies over 2 biological replicate samples, *n* > 20,000 individual LD tracks. Data was analysed using a one-way AVOVA with Dunnett’s multiple comparisons test ** = *p* < 0.01, ns = not significant.

To visualise the cell-to-cell variation in data across multiple data sets, we initially constructed scatter plots with all cells from each FoV. As can be seen in Figure 2a, there was little cell-to-cell variation when comparing the mean LD speed within 16 chosen analysed ROIs from two conditions. However, there was a noticeable shift between the two different conditioned groups graphed (Figure 2a). As there were >40 cell ROIs analysed for each condition, this data was further condensed for multi-cell analysis (Figure 2b). In this case, each data point represents all LD tracks within a cell’s ROI. Figure 3, Figure 4, Figure 5 and Figure 6 are therefore represented as multi-cell analysis, represented as box and whisker plots showing the mean, with 25–75 percentile range as the box and 5–95 percentiles as the whiskers.

### 2.3. Lipid Droplet Motility Is Increased Following Activation of Innate Immune Receptors

It is well-established that viruses can usurp host lipids to fuel their replication [30,31,32]; therefore, we first investigated LD dynamics following stimulation of astrocyte cells with the synthetic dsRNA viral mimic, poly I:C, known to mimic viral RNA pathogen-associated molecular patterns (PAMPs), and activate the RNA sensors RIG-I and TLR-3 to drive an immune response (reviewed in [33]). In order to decipher the change in LD dynamics in astrocytes, we first optimised the delivery of a fluorescently labelled poly I:C (dsRNA viral mimic) into cells, to achieve at least a 95% uptake of the RNA (Figure 3a). Following stimulation with the dsRNA viral mimic, live-stained LDs within astrocyte cells were tracked over a time course from 2 h post-infection up to 72 h, and the average displacement and speed of LDs was analysed (Figure 3b–d). We observed four main diffusion types of LDs: tight restricted, restricted, directed, and random (Figure S1 and Video S1). As early as 4 h post-dsRNA stimulation, LDs were consistently more motile (Figure 3b–d) and were observed to frequently travel in a directed manner, changing paths multiple times (Figure 3b). There was a small, but significant increase in the displacement of LDs at 2 h post-stimulation; however, this was not associated with an increase in speed (Figure 3c,d). LD displacement was significantly increased at all timepoints, with the peak of displacement occurring at 48 h post-stimulation, with an average displacement of 0.85 μm, and some individual cell averages reaching up to 1.41 μm within this timepoint data set (Figure 3c). The average speed of LDs following viral mimic stimulation was increased across all timepoints with the exception of the 2 h timepoint. The average speed of LDs peaked at 24 h post-stimulation, with an average speed of 0.034 μm/sec (Figure 3d); and this was maintained throughout the time course. However, at 8 h post-dsRNA stimulation, there was a significant dip in the average displacement and speed of LDs (Figure 3c,d). The detection of dsRNA by sentinel innate immune receptors initiates a signalling cascade that results in the rapid production of interferon, and it is possible that the dip in LD speed and displacement following 8 h is lifted via the production of interferon (IFN).

### 2.4. Lipid Droplet Motility Is Increased Following IFN-β Stimulation

Following activation of immune receptors by foreign dsRNA, the cell upregulates an antiviral response culminating in the production of antiviral cytokines; most notably type I IFN, IFN-α/β [33]. IFNs are then secreted by the infected cell, to activate a secondary immune pathway in neighbouring cells, priming them before infection (reviewed in [34]). We have previously demonstrated that IFN responses can be altered when LD dynamics are changed [24], and we therefore wanted to assess if IFN stimulation would impact LD motility in vitro. Astrocytes in culture were stimulated with the type-I IFN, IFN-β, and LD dynamics were tracked (Figure 4). Average LD displacement and speed were significantly increased from the control at all timepoints following stimulation and peaked at 8 h post-stimulation (Figure 4a,b). The average LD displacement and speed within cells reached levels greater than those seen following dsRNA treatment, peaking at 0.99 μm and 0.044 μm/s. 

### 2.5. Zika Virus Infection Drives an Increase in Lipid Droplet Mean Speed and Displacement

It is clear that LD dynamics change following synthetic activation of the innate immune pathways; however, to determine whether this also occurred in the context of a true viral infection, we infected astrocyte cells with an RNA virus, Zika virus (ZIKV), and monitored LD dynamics over three timepoints (Figure 5). Following live LD tracking, cells were fixed and stained for viral RNA. A spreading viral infection was observed, with an average cell infection rate of 32% at the initial timepoint, with all cells displaying viral RNA by 24 h (Figure 5a). When assessing the LD dynamics of these cells, a similar trend was observed where there was an increased average displacement and speed of LDs post-ZIKV infection (Figure 5b,c). LD displacement following ZIKV infection peaked at 24 h, with an average of 1.01 μm distance travelled by LDs, with some LDs averaging a displacement of 1.46 μm (Figure 5b). In contrast, the average speed of the LDs peaked at 8 h post-infection, with LDs travelling at 0.044 μm/s on average; however, the fastest LDs were seen at the 24 h timepoint and reached 0.064 μm/s, with the average of this timepoint being lower at 0.041 μm/s (Figure 5c).

### 2.6. Increased Lipid Droplet Motility Is Not Due to an Increase in Lipid Droplet Numbers

It has been well described by us and others that pathogen infection of cells can drive an increase in the number of LDs in a cell [22]. To assess if simply increasing the number of LDs per cell could drive a change in LD dynamics, astrocytes were treated with oleic acid (OA), a long-chain fatty acid (C18:1) that has been demonstrated to stimulate the biogenesis of LDs in different cell types including astrocytes [35]. Cells were treated with 500 µM of OA in media 16 h prior to being imaged and LD dynamics tracked. OA drove a large increase in the number of LDs in the astrocytes (Figure 6a); however, we saw only a small but significant decrease in the distance travelled by these LDs in comparison to the vehicle-only treated cells (Figure 6b), with a lot more LDs within the OA-treated group being tightly restricted (Figure 6a). Interestingly, although we saw an average decrease in displacement of LDs in the OA-treated cells, there was no significant change in the speed of these LDs (Figure 6c). This data suggests that the number of LDs alone has little impact on the changing LD dynamics we observe following infection.

## 3. Discussion

Organelle movement within the cell cytoplasm is not a new phenomenon, and recent research has shown that it is critical for the transfer of proteins and lipids between organelles during cellular events. All organelles within cells are transported and actively positioned to maintain correct cellular organisation and effective cell functioning. LDs specifically have been described to move in two different ways, with ~95% of LDs undergoing diffusive motions [36]. As LD motility alters the spatial position of LDs relative to the rest of the cell and presumably to other organelles, it is expected to have a profound effect on establishing and breaking LD–organelle contacts, and therefore on the flux of lipids through the cell. LDs have been well described to interact with other organelles within the cell to serve as the source of lipids for membrane expansion, energy production and signalling [11,37]. During viral infection, LDs have been demonstrated to be redistributed within the cell, and this has been hypothesised to be associated with viral host cell rearrangement, in order to facilitate a more effective viral life cycle [19,20]. However, it has recently been demonstrated that LDs are upregulated very early following both viral and bacterial infection, likely prior to pathogen replication [21] (reviewed in [22]). The dynamics such as speed and displacement of LDs remains unstudied in the context of very early viral infection; here, we demonstrate that LD dynamics significantly change following the activation of antiviral innate immune receptors. 

To determine if viral infection could change LD dynamics within a cell, a methods pipeline was developed to track individual LDs within cells before and following viral infection (Figure 1). The TrackMate plugin was used to extract the mean speed and displacement of LDs within a cell, and there was a significant enhancement of both these parameters following activation of the RNA-sensing early innate immune pathways (Figure 3). As this upregulation was also mimicked by the stimulation of cells with IFN-β and infection with the RNA virus, Zika virus (Figure 4 and Figure 5), we hypothesise that this could be a perpetuating event, where mean LD speed and displacement could be upregulated via activation of innate immune sensors, and then kept upregulated via the production of cellular IFN.

There have been extensive studies in yeast models to unravel how LDs move within cells. It has been described that the directed motion of LDs along linear tracks can be driven by cytoskeletal motors, such as kinesins, dyneins and myosins (reviewed in [2,38]); however, other mechanisms of transport have also been identified, including polymerisation and growth of actin filaments [39], tethering to other organelles such as early endosomes [40], as well as simple Brownian motion [41]. In this study we saw directed, random and tethered LD motion across all treatment conditions and timepoints (Figure 3, Figure S1 and Video S1). The exact drivers and regulators of altered LD movement remain relatively undescribed, especially in mammalian cells. Rab GTPases are known to play large roles in the movement of many organelles intracellularly (reviewed in [42]), and recent research has shown that this family of proteins are highly dynamic in the proteome of mammalian cell LDs [23,43], so may well be involved in orchestrating LD movement; however, further research is required to fully elucidate these mechanisms. 

It has now been described by us and others that LDs accumulate in pathogen-infected cells [21] (reviewed in [22]). LDs are heterogeneous, with a large spread of LD sizes ranging from < 200 to over 5000 nm (reviewed in [44]). We have previously described that there is a significant increase in the number of nascent LDs (< 200 nm) within infected cells; however, the role of different LD populations is unknown. Here, we demonstrate that there is a significant increase in the mean speed and displacement of LDs following viral infection; however, it is important to also note that there seems to be a population of LDs which do not increase their motility during infection (Figure 3, Figure 4, Figure 5 and Figure 6). It is possible that different populations of LDs have alternate roles within the cells, and it has been previously described that LD size and age can dictate altered LD proteomes and the ability to recruit certain proteins [45]; additionally, studies have shown that LD lipid content may also influence the organelle’s proteome [46].

Lipid droplets are well recognised for their ability to interact with other organelles, and an increase in LD speed and distance travelled may well also drive enhanced inter-organellar communication. Organelle–organelle communication is critical for the transfer of proteins and lipids to fuel cellular events, and following detection of invading viral nucleic acid by sentinel innate immune receptors, important adaptor proteins within this pathway come together to form enhansosomes. Multiple proteins involved in the antiviral response have been shown to facilitate enhansosome formation with the LD as a platform, and it is possible that LD movement might facilitate this activity [25,26,47]. Additionally, recent work has demonstrated that LDs move away from mitochondrial contacts following detection of bacterial LPS by sentinel innate immune receptors, towards intracellular bacteria, facilitating a cellular anti-bacterial response, perhaps indicating that LD movement is critical following pathogen infection to ensure a good outcome for the host cell.

We have shown for the first time that LDs alter their dynamics as early as 2 h following viral infection or following activation of early innate signalling pathways. The alterations in LD dynamics are prolonged, and likely to be part of a feedback loop, with interferon production also showing enhanced LD movement and speed, perhaps demonstrating that these dynamics underpin part of a successful host response to pathogen infection. However, further work will be required to elucidate this. The dynamic movement of organelles within a cell following viral infection is understudied and expanding knowledge in this area may assist in targeting host pathways to develop novel anti-viral therapeutics that boost the host cell’s response to an infection. Further research will be required to explain our gaps in knowledge surrounding the driving forces behind the motility of these specialised organelles.

## 4. Materials and Methods

### 4.1. Cells and Culture Conditions

Primary Immortalised Human Astrocytes were maintained in DMEM (Gibco, Waltham, MA, USA) containing 10% fetal bovine serum (FBS), 100 units/mL penicillin and 100 μg/mL streptomycin and were maintained at 37 °C in a 5% CO_2_ air atmosphere. Cells were seeded into glass bottom dishes (Matek, Ashland, DE, USA) at a density of 3 × 10^5^ cells per dish. 

### 4.2. Viral Infection and Viral Mimics

The dsRNA viral mimic, poly I:C (Invivogen, San Diego, CA, USA), was transfected into cells using PEI transfection reagent (Sigma-Aldrich, St. Louis, MO, United States) as per manufacturer’s instructions at a concentration of 1 µg/mL. Control stimulated cells received PEI transfection reagent only. Poly I:C tagged with Rhodamine was used at 1 µg/mL to quantify the percentage of cells stimulated by poly I:C in our experiments. Zika virus (ZIKV MR777 strain) was used to infect primary immortalised astrocyte cells. Cells were washed with phosphate-buffered saline (PBS) at the indicated multiplicity of infection (MOI) in DMEM without FCS. After 2 h of incubation, the infection medium was removed and replaced with DMEM containing 10% (*v*/*v*) FCS. 

### 4.3. Oleic Acid Treatment

To increase the number of LDs in cells, oleic acid (OA) (n-9 MUFA, C18:1)—a long-chain fatty acid—was used as a treatment on cells. OA was purchased from Sigma (Sigma-Aldrich, St. Louis, MO, United States) and dissolved in 0.1% NaOH and 10% bovine serum albumin (BSA). OA was prepared as a 10 mM stock solution and stored at −20 °C until required. BSA was used as a vehicle control. Cells were treated with 500 μM OA in DMEM (+1%BSA) for 6 h.

### 4.4. Live Lipid Droplet Staining

For live staining of LDs, primary immortalised astrocyte cells were cultured on coverslip bottom 35 mm dishes (MatTek) and were incubated with Bodipy 493/503 (1 ng/mL in complete DMEM) for 1 h prior to imaging at 37 °C. Samples were then washed with PBS and given fresh complete DMEM without Bodipy for imaging. 

### 4.5. Fixed Lipid Droplet and Viral Staining

Bodipy (493/503) staining for fixed cells was performed as previously described [24]. Briefly, cells were grown in either 24-well plates on 12 mm glass coverslips coated with 0.2% gelatine or were seeded into 35 mm glass bottom dishes. Cells were washed with 1× PBS, fixed with fresh 4% paraformaldehyde (PFA) in PBS for 15 min at room temperature and permeabilised with 0.1% Triton X-100 in PBS for 10 min. Cells were then blocked with PBS containing 5% BSA (Sigma-Aldrich, St. Louis, MO, United States) for 30 min at room temperature before incubation for 1 h at room temperature with primary antibody diluted in PBS containing 1% BSA. ZIKV was detected using anti-3G1.1 and 2G4 dsRNA hybridoma antibodies neat for 1 h. Cells were then washed and incubated with Alexa Fluor 555 secondary antibody at 1:200 for 1 h. Bodipy (493/503) was used to stain LDs at 1 ng/mL for 1 h at room temperature, and nuclei were stained with DAPI for 5 min at room temperature. 

### 4.6. Image Acquisition

All images were acquired using a Nikon TiE inverted fluorescence microscope. Unless otherwise indicated, images were processed using NIS Elements AR v.3.22. (Nikon, Tokyo, Japan) and FIJI analysis software. During acquisition, cells were maintained at 37 °C using a heated microscope stage insert (Biotherm™) and visualised using a × 60 NA 1.4 oil immersion objective lens (Nikon, Tokyo, Japan). For all of these experiments, a Nikon perfect focus system was applied. Images were acquired (without binning) every 5 s using exposure times of 500 ms and a maximum camera gain setting of 2. 

### 4.7. Image Processing and Analysis

Enumeration of individual RNA foci was performed manually by counting > 300 cells (across 2 biological replicates) positive for the fluorescently tagged dsRNA viral mimic. This analysis was presented as a percentage of stimulated cells. 

For live cell imaging, no contrast or background subtraction was applied to any frames analysed. For each condition and timepoint, 3 FoV movies from 2 biological replicates (*n* = 6 movies from each condition) were acquired with a total of 49 images per movie (500 ms exposure, images captured every 5 s). Image stacks were imported into FIJI analysis software [48] and registered using the linear stack alignment with SIFT (scale invariant feature transform) plugin [28]. For each FoV image, ROIs were drawn around single cell outlines for LD tracking analysis. LD tracking was performed using the ‘TrackMate’ plugin on FIJI analysis software [29] with the following parameters: estimated blob diameter = 0.5 μm and threshold = 500. Simple Linear Assignment Problem (LAP) tracker was used to link together identified tracks with the following parameters: linking max distance = 1 μm, gap-closing max distance = 1 μm and gap-closing max frame gap = 0 μm, to ensure no false positive tracks were linked. Data was extracted for mean speed, displacement and track quality. Extracted data was then imported into Excel for clean-up. Data was further excluded if large cell morphology changes (i.e., dying cells) were observed and correlated with large-scale, uniform LD movements, or if tracks were less than 10 frames in total length.

### 4.8. Statistical Analysis

Data is represented as multi-cell analysis, where each data point is an average of all of the LD tracks within a single cell. Box and whisker plots showing the mean, with 25–75 percentile range as the box and 5–95 percentiles as the whiskers, demonstrate the mean speed and displacement of the data. Statistics were calculated using a one-way AVOVA with Dunnett’s multiple comparisons test, with *p* < 0.05 considered to be significant. All statistical analysis was performed using Prism 9 (GraphPad Software, San Diego, CA, USA).

## Data Availability

The data presented in this study are openly available in FigShare at https://figshare.com/projects/Lipid_Droplet_Motility/101420.

## Supplementary Materials

The following are available online at https://www.mdpi.com/article/10.3390/ijms22094418/s1, Figure S1: Lipid droplet diffusion types, Video S1: Lipid droplet tracks 4 h post-dsRNA stimulation.
