# Supplementary material for: Lipid Droplet Motility Increases Following Viral Immune Stimulation
Source: Int J Mol Sci. 2021 Apr 23;22(9):4418. doi: 10.3390/ijms22094418 (PMC8122965; doi:10.3390/ijms22094418)
Supplement: Supplementary file 1 [file ijms-22-04418-s001.zip › ijms-1185564-supplementary.pdf]

International Journal of *Molecular Sciences*

MDPI

Supplementary Material

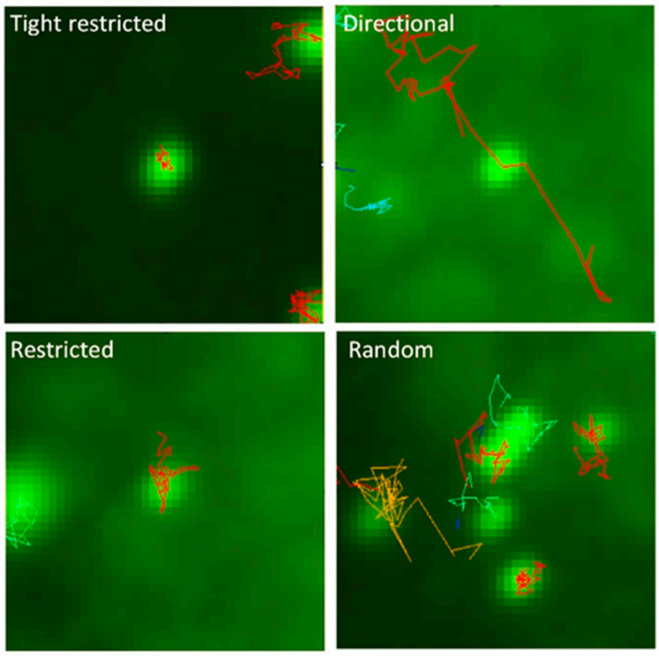

Figure S1. Lipid droplet diffusion types. There were 4 different diffusion types of LDs observed in the study; tight restricted, restricted, directional and random.

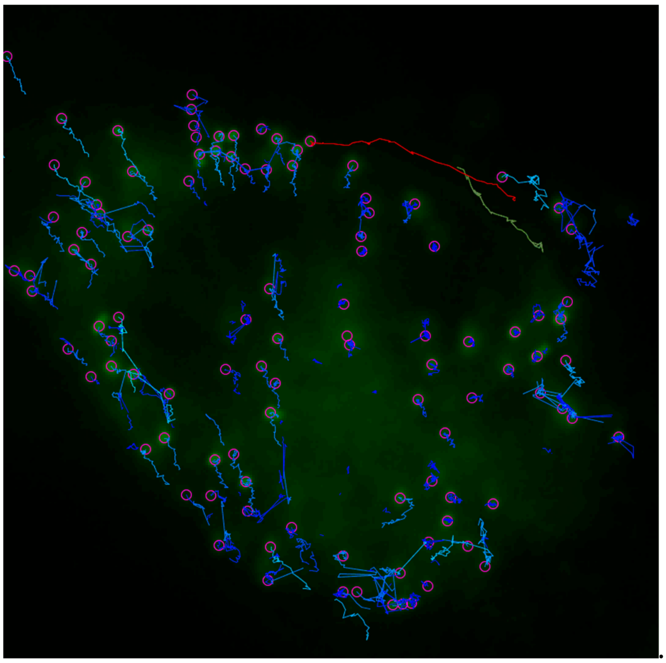

Video S1. Lipid droplet tracks 4 h post-dsRNA stimulation. Images were captured every 5 s for a total of 49 frames. The TrackMate software on FIJI was used to track individual LDs within cells.
